# Scombroid Poisoning with Concurrent *Brevundimonas* Septicemia: A Unique Case Report and Brief Literature Review

**DOI:** 10.1155/2019/2148654

**Published:** 2019-11-11

**Authors:** Caroline C. Eskind, Cassandra A. Doucet, Bryan D. Harris

**Affiliations:** ^1^Division of Infectious Diseases, Department of Medicine, Vanderbilt University Medical Center, Nashville, TN 37232, USA; ^2^Department of Medicine, Vanderbilt University Medical Center, Nashville, TN 37232, USA

## Abstract

Scombroid poisoning is a predominantly self-limited illness associated with ingestion of poorly handled fish. It is not frequently associated with bacteremia and has never been described with *Brevundimonas* septicemia. We describe a case of a man who presented in shock with histamine poisoning after ingesting sushi. Blood cultures grew an uncommon pathogen, *Brevundimonas vesicularis*. This case demonstrates systemic bacterial infection in the setting of histamine poisoning, which is an atypical presentation for a well-known foodborne illness.

## 1. Background

Scombroid poisoning is a relatively infrequent cause of foodborne toxicity that is usually attributable to ingestion of improperly handled fish. While most commonly identified in association with fish of the Scombridae family, such as mackerel, albacore, and bonito, poisoning has been attributed to ingestion of other fish as well, including mahi-mahi, salmon, and sardines. Symptoms are due to ingestion of histamine which is produced on the surface of improperly stored fish. Symptoms include flushing, vomiting, diarrhea, urticaria, and occasionally more severe symptoms such as cardiopulmonary distress and shock. A variety of bacteria have been implicated in the reaction to convert histidine to histamine through the enzyme histidine decarboxylase, including *Proteus*, *Enterobacter*, *Serratia*, *Pseudomonas,* and *Vibrio*. Treatment primarily consists of antihistamines and supportive care, and symptoms usually self-resolve in under 12 hours [1, 2]. Bacteremia has not been routinely described as a complication of scombroid poisoning, though affected patients can be severely ill with shock, myocardial infarction, or respiratory distress with bronchospasm [1].

*Brevundimonas vesicularis* is a motile Gram-negative bacillus, previously identified as a member of the *Pseudomonas* family. It is commonly found in soil, as well as a variety of aqueous environments including bottled and filtered water, pools, and deep-sea water [3–5]. By 2017, only 35 cases of human infection with *B. vesicularis* had been described in the literature, including cases of bacteremia, endocarditis, peritonitis, and urinary tract infections [4]. Infected patients are usually immunocompromised with malignancy, autoimmune disease, or multiple comorbid conditions, such as diabetes and end-stage renal disease. Isolates have variable sensitivity profiles, but regularly are resistant to ampicillin and fluoroquinolones and occasionally to 1^st^ and 2^nd^ generation cephalosporins [3–5]. Cases are described as community-acquired or hospital-acquired, but the original source of the bacteria is rarely identified and has never been associated with fish consumption [6].

We report a case of an immunocompetent man who was admitted to an acute care hospital in shock, with presumed scombroid poisoning, who was later found to have concurrent bacteremia with *B. vesicularis*.

## 2. Case Presentation

A 76-year-old man with chronic diastolic heart failure, well-controlled bipolar disorder on lithium, and atrial fibrillation was admitted to the hospital with gastrointestinal distress that developed just six hours prior to admission. He had been briefly hospitalized less than two weeks prior due to a heart failure exacerbation requiring intravenous (IV) diuretics, but was feeling well in the days leading up to presentation. For dinner that evening, he and his wife consumed prepared sushi containing imitation crab meat that had been purchased the day before from a national grocery store chain. Within 45 minutes of consumption, he developed sudden nausea, violent emesis, and nonbloody diarrhea. He developed diffuse pruritus and discovered an urticarial rash covering the majority of his skin. He took two tablets of Benadryl and retired to his bedroom, where he eventually fell onto the floor and was overwhelmingly weak, unable to stand or walk. Per EMS report, he was hypotensive to 50 s/30 s and was given 1 liter of IV fluids en route to the hospital. Upon arrival to the hospital, he remained hypotensive to 70 s/40 s and tachycardic to 120 s, but his gastrointestinal symptoms and rash had already resolved. He required admission to the intensive care unit due to persistent hypotension despite fluid resuscitation, and he was given intravenous norephinephrine. He was given 1000 mg IV vancomycin every 12 hours and 3.375 grams IV piperacillin-tazobactam every 6 hours for presumed septic shock and was also given IV Benadryl and subsequently switched to oral cetirizine for presumed histamine fish toxicity. His shock resolved by the next day after receiving 5 L of IV fluids, and the vasopressor was weaned off.

Initial lab tests were significant for a leukocyte count of 24,000 cells/*μ*L, potassium of 6.4 mEq/L, serum creatinine of 2.3 mg/dL, lactic acid of 4.1 mmol/L, troponin of 0.05 ng/L, and brain-type natriuretic peptide of 3730 pg/mL. Of note, a noncontrasted computerized tomography (CT) scan obtained on admission was concerning for sigmoid colitis. The aerobic bottle of his blood cultures (BD Bactec) obtained upon admission eventually grew a Gram-negative bacillus, which was later identified as *B. vesicularis* using Vitek 2 identification with the VITEK 2 GN ID card. Antibiotic susceptibilities were determined using the Vitek 2 system with AST-GN69 and AST-XN06 cards to determine mean inhibitory concentration (MIC) based on the Clinical and Laboratory Standards Institute (CLSI) guidelines. The isolate was sensitive to piperacillin-tazobactam, amikacin, tobramycin, gentamicin, imipenem, and tetracycline and was resistant to fluoroquinolones, aztreonam, and ceftazidime. The public health department was notified and, unfortunately, the sushi had already been consumed and disposed of, so it was not available for testing. A urine methylhistamine level was ordered, but ultimately, the testing was never performed by the lab.

He remained on renally dosed IV piperacillin-tazobactam at 2.25 grams every 6 hours and initially improved with antibiotics and supportive care, and subsequent blood culture bottles did not grow any further bacteria. Later in his hospital course, he decompensated with progressive renal failure, acute liver injury, and persistent leukocytosis. He underwent repeat CT scanning, which demonstrated a small amount of pericholecystic fluid and small dependent gallbladder calculi. His previously described sigmoid colitis had improved radiographically. These findings were thought to represent acute cholecystitis or secondary liver disease in the setting of worsening abdominal ascites. He was transferred back to the ICU due to tachycardia to the 160–170 s and hypotension.

Cardiology was consulted and attributed his clinical decompensation to heart failure with congestive hepatopathy. Despite broad-spectrum antibiotics, vasopressor support, and diuretics, his clinical status did not improve and his family elected to pursue comfort measures. He passed away in the ICU on hospital day 10.

## 3. Discussion

*Brevundimonas vesicularis* is infrequently described in cases of human infection, most often affecting patients with underlying immunocompromising conditions. The source of bacterial infection is thought to be community-derived in most cases; however, the organism itself is frequently isolated from water sources [6]. Cases have been reported in the bloodstream of patients acutely postoperative from cardiac surgery and in meningeal infection after surgical resection of a brain tumor. Patients have also been diagnosed with *B. vesicularis* peritonitis in the setting of peritoneal dialysis [4]. These infections raise the possibility of the bacteria being introduced into patients by contaminated fluid. Notably, *Brevundimonas* species are very small and can pass through sterilizing filters and have been identified in bottled water and in cases of pseudooutbreaks that truly represented contamination of laboratory media [4]. This did not appear to be the case in our laboratory, as the isolate was not identified in any other specimens.

We hypothesize that our patient may have acquired infection with *Brevundimonas* from consumption of improperly stored fish that lead to simultaneous histamine poisoning and bacteremia. Unfortunately, the food source was not kept and, therefore, confirmatory testing was not possible. *Brevundimonas* spp. have been isolated from the surface and intestine of various fish [7]; however, they have not frequently been identified as causative agents of histamine poisoning. One study has demonstrated that *Brevundimonas vesicularis* isolated from Spanish mackerel was histamine-forming [8]. *Pseudomonas* species have frequently been associated with histamine production [2]; given the taxonomic relatedness of the two species, it is plausible that *Brevundimonas* spp. share this trait. While no human infection with *Brevundimonas* has been described in relation to seafood consumption, other cases of bloodstream infection with water-associated bacteria, such as *Edwardsiella tarda* and *Raoultella planticola*, have been documented in relation to the consumption of fish [9, 10]. Two cases of septicemia with *Raoultella* spp. have been described with concurrent symptoms of histamine poisoning, but in both cases, definitive testing for histamine toxicity was not performed [11, 12]. Our patient's multidrug resistant isolate is consistent with the existing reports of *Brevundimonas* infections [4, 5]. Our lab was able to identify the bacteria using the advanced colorimetry technique of VITEK 2; however, given the slow-growing nature and infrequent incidence of infection, 16S rRNA sequencing may aid in identification of more cases of *B. vesicularis* infection.

Future cases of *Brevundimonas* infection should be investigated for association with seafood consumption. Similarly, patients with severe manifestations of scombroid poisoning should have blood cultures obtained to identify possible concurrent bacteremia with waterborne pathogens.

## Abbreviations

CT: Computed tomography
EMS: Emergency medical services
ICU: Intensive care unit
IV: Intravenous
rRNA: Ribosomal ribonucleic acid.
